# Localization of myoglobin in mitochondria: implication in regulation of mitochondrial respiration in rat skeletal muscle

**DOI:** 10.14814/phy2.14769

**Published:** 2021-03-02

**Authors:** Rikuhide Koma, Tsubasa Shibaguchi, Claudia Pérez López, Toshihiko Oka, Thomas Jue, Hisashi Takakura, Kazumi Masuda

**Affiliations:** ^1^ Graduate School of Human and Socio‐Environmental Studies Kanazawa University Ishikawa Japan; ^2^ Institute of Liberal Arts and Science Kanazawa University Ishikawa Japan; ^3^ Faculty of Human Sciences Kanazawa University Ishikawa Japan; ^4^ Department of Life Science Rikkyo University Tokyo Japan; ^5^ Department of Biochemistry and Molecular Medicine University of California Davis Davis CA USA; ^6^ Faculty of Health and Sports Science Doshisha University Kyoto Japan

**Keywords:** myoglobin, proteinase K, skeletal muscle, submitochondrial localization

## Abstract

Mitochondria play a principal role in metabolism, and mitochondrial respiration is an important process for producing adenosine triphosphate. Recently, we showed the possibility that the muscle‐specific protein myoglobin (Mb) interacts with mitochondrial complex IV to augment the respiration capacity in skeletal muscles. However, the precise mechanism for the Mb‐mediated upregulation remains under debate. The aim of this study was to ascertain whether Mb is truly integrated into the mitochondria of skeletal muscle and to investigate the submitochondrial localization. Isolated mitochondria from rat gastrocnemius muscle were subjected to different proteinase K (PK) concentrations to digest proteins interacting with the outer membrane. Western blotting analysis revealed that the PK digested translocase of outer mitochondrial membrane 20 (Tom20), and the immunoreactivity of Tom20 decreased with the amount of PK used. However, the immunoreactivity of Mb with PK treatment was better preserved, indicating that Mb is integrated into the mitochondria of skeletal muscle. The mitochondrial protease protection assay experiments suggested that Mb localizes within the mitochondria in the inner membrane from the intermembrane space side. These results strongly suggest that Mb inside muscle mitochondria could be implicated in the regulation of mitochondrial respiration via complex IV.

## Graphical abstract

Recently, we showed the possibility that the muscle‐specific protein myoglobin (Mb) interacts with mitochondrial complex IV to augment the respiration capacity in skeletal muscles. However, the precise mechanism for the Mb‐mediated upregulation remains under debate. Our mitochondrial protease protection assay experiments revealed that Mb localizes within the mitochondria in the inner membrane from the intermembrane space side, strongly suggesting that Mb inside muscle mitochondria could be regulating mitochondrial respiration via complex IV.

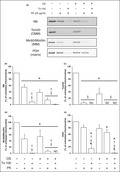

**Abbreviations**
AP: alkaline phosphatase
ATP: adenosine triphosphate
Co‐IP: co‐immunoprecipitation
COX‐IV: cytochrome c oxidase subunit IV
Cyt c: cytochrome c
ETC: electron transport chain
IMM: inner mitochondrial membrane
IMS: intermembrane space
Mb: myoglobin
Mic60/Mitofilin: MICOS complex subunit Mic60
O_2_: oxygen
OMM: outer mitochondrial membrane
OS: osmotic shock
PDH: pyruvate dehydrogenase
PK: proteinase K
SDS: sodium dodecyl sulfate
Tom: translocase of outer mitochondrial membrane
TOM: translocase of the outer membrane
Tx‐100: Triton X‐100

## 1 INTRODUCTION

Mitochondria are essential organelles for cellular energy metabolism, and their respiration is an important process that requires oxygen (O_2_) to convert the energy stored in macronutrients into adenosine triphosphate (ATP). The molecular machinery required for mitochondrial respiration is the electron transport chain (ETC), which is an assembly of electron donors and acceptors (Yu et al., 2013). The ETC mainly consists of four complexes, complex I, II, III, and IV, which are embedded in the inner mitochondrial membrane (IMM). O_2_ serves as a substrate for complex IV, the terminal oxidase of the ETC, during oxidative phosphorylation.

Myoglobin (Mb), a member of the globin superfamily, is mainly expressed in oxidative skeletal muscle myofibers and cardiomyocytes (Kanatous & Mammen, 2010; Ordway & Garry, 2004). Since Kendrew et al. (1958, 1960) determined the three‐dimensional structure of Mb, many studies have been focused on its roles in O_2_ storage, buffering intracellular O_2_ concentrations, and facilitation of O_2_ diffusion (Ordway & Garry, 2004). Mb is treated as cytoplasmic protein in reviews of Mb biochemistry (Kanatous & Mammen, 2010; Ordway & Garry, 2004; Postnikova & Shekhovtsova, 2018). However, several previous studies have suggested that Mb is not only localized in the cytosol but is also closely associated with mitochondria. Taylor et al. (2003) reported in a human heart mitochondrial proteome study that Mb was identified in isolated mitochondria, and Mb was released by washing mitochondria with 150 mM KCl, suggesting that Mb is located on the surface of the outer mitochondrial membrane (OMM). Subsequently, two studies revealed that Mb interacts with the OMM during heme uptake by apomyoglobin and its deoxygenation (Postnikova et al., 2009; Vernier et al., 2007). Interestingly, in skeletal muscle, we have also shown that Mb is co‐localized in mitochondria using western blotting, immunohistochemistry, and electron microscopy (Yamada et al., 2013). In addition, a co‐immunoprecipitation (Co‐IP) analysis revealed that a portion of the Mb present in muscle binds cytochrome c oxidase subunit IV (COX‐IV), a component of mitochondrial complex IV at the IMM. These findings suggest that Mb would be integrated into the mitochondria and would interact with complex IV in muscle cells. Furthermore, we found that overexpression of Mb in cultured rodent muscle cells improved mitochondrial respiration through up‐regulation of complex IV activity, suggesting that Mb inside mitochondria might directly regulate respiration by interacting with complex IV and augmenting its activity (Yamada et al., 2016). However, questions remain about the precise mitochondrial localization of Mb. Differential centrifugation alone cannot remove proteins anchored in the OMM (Yamada et al., 2013), and previous studies cannot confidently exclude possible Mb interactions at the OMM (Postnikova et al., 2009; Taylor et al., 2003; Vernier et al., 2007), which would alter the interpretation of Co‐IP results indicating Mb binding to COX‐IV. Even if Mb is integrated into mitochondria, the submitochondrial localization remains unclear. However, the observation that Mb interacts with the IMM protein COX‐IV (Yamada et al., 2013) suggests localization either in the intermembrane space (IMS) side or the matrix side of the IMM. More detailed knowledge of the submitochondrial localization is needed to clarify the precise mechanism underlying the role of Mb in the regulation of mitochondrial respiration. Therefore, this study aimed to investigate whether Mb is truly integrated into the mitochondria of skeletal muscle and its submitochondrial localization.

## 2 MATERIALS AND METHODS

### 2.1 Ethical approval

All experimental procedures were conducted under the Guide for the Care and Use of Laboratory Animals of the Physiological Society of Japan. This study was also approved by the Ethics Committee on Animal Experimentation of Kanazawa University (Protocol #: AP‐10187).

### 2.2 Animals

Adult male Wistar rats (282–375 g) were obtained from Japan SLC Corporation. Experimental animals were housed in an air‐conditioned room under laboratory environmental conditions (12:12 light/dark cycle; room temperature, 23 ± 2°C; humidity, 55 ± 5%). A standard diet (MF; Oriental Yeast) and water were provided *ad libitum*.

### 2.3 Sampling

Animals were anesthetized with medetomidine (0.3 mg/kg‐BW *i*.*p*.; Nippon Zenyaku Kogyo Co., Ltd.), midazolam (4 mg/kg‐BW *i*.*p*.; Maruishi Pharmaceutical Co., Ltd.), and butorphanol (5 mg/kg‐BW *i*.*p*.; Meiji Seika Pharma Co., Ltd.). The gastrocnemius muscles of both hindlimbs were removed and washed in ice‐cold saline. After removing the connective tissue, fat, and nerve, the muscles were weighed, clamp‐frozen in liquid nitrogen, and then stored at −80°C until subsequent analyses.

### 2.4 Preparation of mitochondria

**FIGURE 1:**
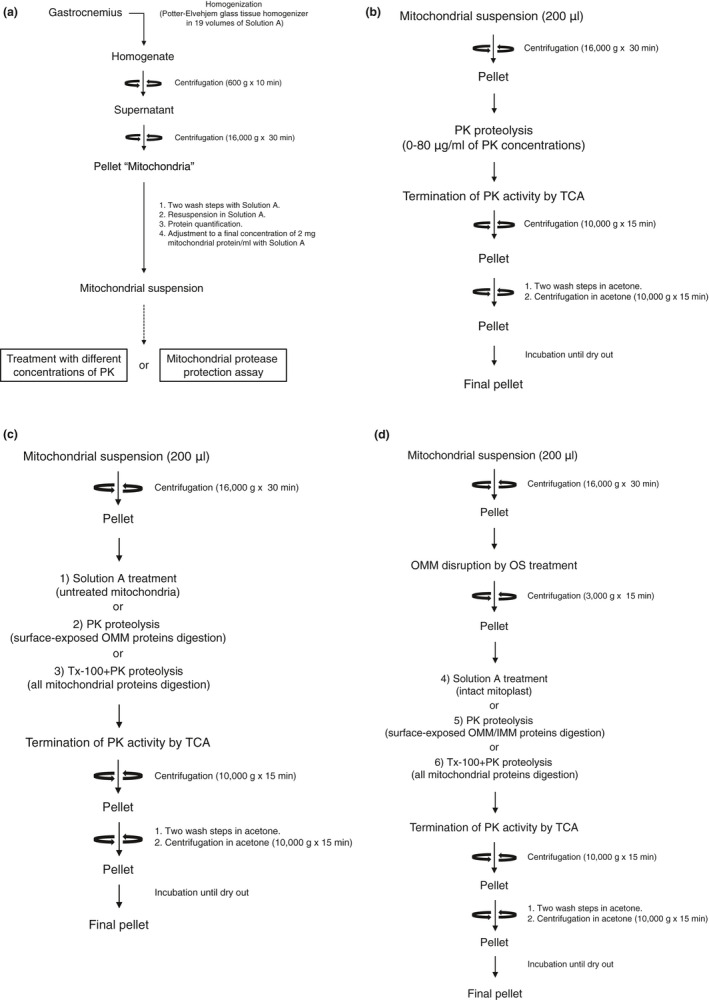
Schematic representation of biochemical approaches in the present study. (a) Scheme for isolating mitochondria from rat gastrocnemius. (b) Scheme for performing the treatment with different PK concentrations. (c–d) Scheme for performing the mitochondrial protease protection assay. IMM, inner mitochondrial membrane; OMM, outer mitochondrial membrane; OS, osmotic shock; PK, proteinase K; TCA, trichloroacetic acid; and Tx‐100, Triton X‐100.

Crude mitochondria were isolated from a deep portion of the gastrocnemius muscle according to the modified method of Hashimoto et al. (2006) and van Vlies et al. (2007). Briefly, the tissue was homogenized in 19 volumes of ice‐cold Solution A (250 mM sucrose, 5 mM NaN_3_, 2 mM EGTA, 20 mM HEPES‐Na, pH 7.4) with thirty strokes of a Teflon pestle in a Potter‐Elvehjem glass tissue homogenizer at 1,000 rpm. The homogenate was centrifuged at 600 *g* for 10 min at 4°C to remove nuclei and debris. The supernatant was further centrifuged at 16,000 *g* for 30 min at 4°C, and the pellet was collected as the crude mitochondrial fraction. The crude mitochondrial pellet was washed twice in Solution A and then re‐suspended in Solution A. The protein concentration of the re‐suspension was determined by the method of Bradford (1976), using a protein assay kit (Bio‐Rad Laboratories). The crude mitochondrial re‐suspension was adjusted to a final concentration of 2 mg mitochondrial protein/ml with Solution A and then used for the following assays (Figure 1a).

### 2.5 Treatment with different concentrations of proteinase K

Treatment with different concentrations of proteinase K was performed according to the modified method of Tammineni et al. (2013). The crude mitochondrial suspension was divided into six equal aliquots of 200 µl and were centrifuged at 16,000 *g* for 30 min at 4°C. Since few studies have examined the appropriate PK concentration in detail for skeletal muscle mitochondria, we have set the PK concentration to 0–80 μg/ml based on the previous studies on other cell types (Izumikawa et al., 2017; Tammineni et al., 2013). The pellets were re‐suspended in 500 µl of Solution A containing proteinase K (PK; P2308; Sigma‐Aldrich) at a final concentration of 0–80 µg/ml and incubated on ice for 10 min. After incubation, an equal volume of 20% (w/v) trichloroacetic acid (TCA) in Solution A was added to each tube and incubated on ice for 15 min to terminate the PK activity. All six tubes were then pelleted by centrifugation at 10,000 *g* for 15 min at 4°C. The resultant mitochondrial pellets were washed twice with acetone and centrifuged at 10,000 *g* for 10 min at 4°C. After centrifugation, the pellets were air‐dried at room temperature for 60 min to completely remove the acetone from the pellet (Figure 1b). The final pellets were solubilized in 400 µl of sodium dodecyl sulfate (SDS) sample buffer. The samples were incubated at 95°C for 5 min and then used for western blotting.

### 2.6 Mitochondrial protease protection assay

Mitochondrial protease protection assay was performed according to the modified method of Badugu et al. (2008). The crude mitochondrial suspension was divided into six equal aliquots of 200 µl and were centrifuged at 16,000 *g* for 30 min at 4°C. Three mitochondrial pellets were subjected to the following treatments: (1) a pellet was re‐suspended in 500 µl of Solution A and incubated on ice for 10 min (untreated mitochondria); (2) a pellet was re‐suspended in 500 µl of Solution A containing 20 µg/ml of PK and incubated on ice for 10 min (surface‐exposed OMM proteins’ digestion); and (3) a pellet was re‐suspended and incubated with 400 µl of 1% (v/v) Triton X‐100 (Tx‐100) in Solution A on ice for 20 min and then incubated with 500 µl of Solution A containing 20 µg/ml of PK on ice for 10 min (all mitochondrial proteins’ digestion). The other three were subjected to osmotic shock (OS) by re‐suspension in 250 µl of a hypotonic solution (5 mM HEPES, 5 mM sucrose, 1 mM EGTA, pH 7.4) and then incubated on ice for 10 min to disrupt the OMM. After incubation, an equal volume of a hypertonic solution (750 mM KCl, 80 mM HEPES, 1 mM EGTA, pH7.4) was added to the three tubes to re‐establish isotonic conditions. The suspension was centrifuged at 3,000 *g* for 15 min at 4°C to obtain mitoplasts (pellet). The supernatant was further centrifuged at 220,000 *g* for 60 min, and the final supernatant was collected as the IMS fraction and the remaining pellet contained fragments of OMM and IMM. The three mitoplast pellets were subjected to the following treatments: (4) a pellet was re‐suspended in 500 µl of Solution A and incubated on ice for 10 min (intact mitoplast); (5) a pellet was re‐suspended in 500 µl of Solution A containing 20 µg/ml of PK and incubated on ice for 10 min (surface‐exposed OMM/IMM proteins’ digestion); and (6) a pellet was re‐suspended and incubated with 400 µl of 1% (v/v) Tx‐100 in Solution A on ice for 20 min and then incubated with 500 µl of Solution A containing 20 µg/ml of PK on ice for 10 min (all mitochondrial proteins’ digestion). Following the last incubation of each treatment, TCA precipitations of each treatment were collected and solubilized with 400 µl of SDS sample buffer using the same process as for the PK treatments described above (Figure 1c,d).

### 2.7 Western blotting

Western blot analysis was performed according to the modified method of Yamada et al. (2013). Proteins were resolved by 12–16% SDS polyacrylamide gel electrophoresis, and separated proteins were electrophoretically transferred onto polyvinylidene difluoride membranes (Clear Blot Membrane‐P plus; ATTO, Tokyo, Japan) using a semi‐dry system (WSE‐4045 HorizeBLOT 4 M; ATTO). The membranes were washed with Tris‐buffered saline (150 mM NaCl, 25 mM Tris‐HCl, pH 7.4) containing 0.1% (v/v) Tween‐20 (TBS‐T) for 10 min and blocked with 4% (w/v) Block Ace [for translocase of outer mitochondrial membrane 20 (Tom20)] or TBS‐T containing 5% (w/v) skim milk (for other proteins) at room temperature for 1 h. The membranes were then incubated with rabbit polyclonal antibodies against pyruvate dehydrogenase (PDH; 1:2,000; 18068‐1‐AP; Proteintech), Mb (1:1,000; sc‐25607; Santa Cruz Biotechnology), and Tom20 (1:1,000; sc‐11415; Santa Cruz Biotechnology), and mouse monoclonal antibody against cytochrome c (Cyt c; 1:5,000; sc‐13156; Santa Cruz Biotechnology) and MICOS complex subunit Mic60 (Mic60/Mitofilin; 1:1,000; ab110329; Abcam) at room temperature for 1 h. These antibodies were diluted in TBS‐T containing 5% (w/v) bovine serum albumin and 0.02% (w/v) NaN_3_. After three washes with TBS‐T, the membranes were reacted with alkaline phosphatase (AP)‐conjugated anti‐rabbit IgG (1:3,000; #7054; Cell Signaling Technology) or anti‐mouse IgG secondary antibody (1:3,000; #7056; Cell Signaling Technology) in 4% (w/v) Block Ace or TBS‐T containing 5% (w/v) skim milk for 1 h at room temperature. Following three washes with TBS‐T, protein signals were visualized by the chemiluminescence detection method using the Immune‐Star^TM^ AP Chemiluminescence Kit (Bio‐Rad Laboratories) and captured with MicroChemi (Berthold Technologies). Immunoreactivities were quantified using the Image J software (NIH). Immunoreactivity of untreated mitochondria was set as 100%.

### 2.8 Statistical analysis

All data are presented as the mean ± SD. One‐way ANOVA was used to evaluate western blot data for treatments with different PK concentrations and mitochondrial subfractionation. When a significant difference was revealed, Bonferroni's post hoc test was conducted. Differences in mean values between untreated and OS treated mitochondria were tested by an unpaired *t* test. The significance was set at *p* < 0.05.

## 3 RESULTS

### 3.1 Treatment with different concentrations of PK

**FIGURE 2:**
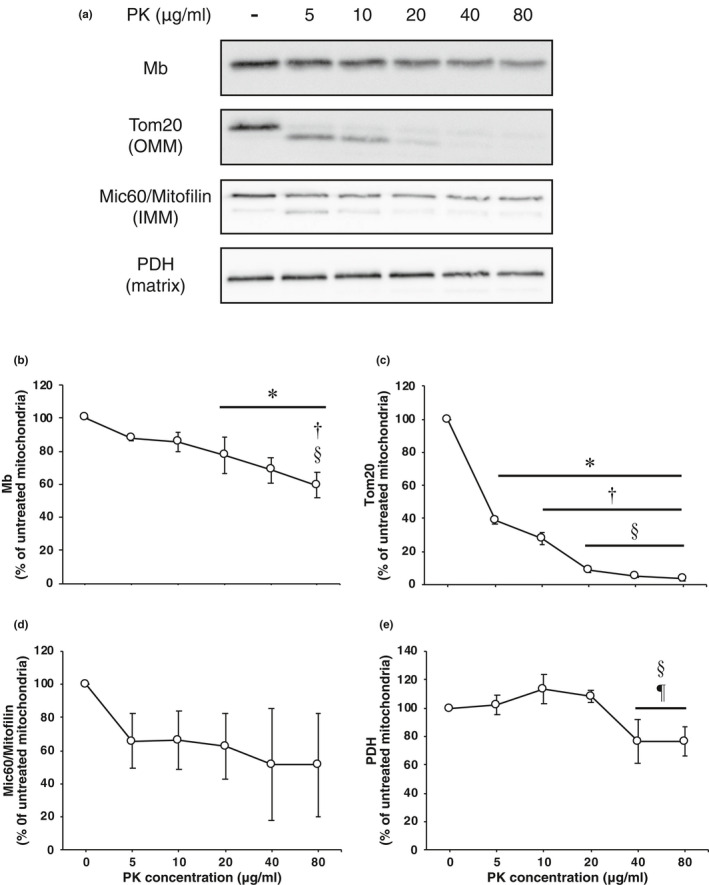
Mb is localized inside the mitochondria of skeletal muscle. (a) Western blotting was performed with antibodies for Mb, Tom20, Mic60/Mitofilin and PDH on isolated mitochondria treated with different PK concentrations. (b–e) Quantification of the immunoreactivities of all analyzed proteins in PK treated mitochondria (*n* = 3 from separate rats). The immunoreactivities of proteins of untreated mitochondria were set as 100%. The values are means ± SD. Significant differences were assessed using one‐way ANOVA and Bonferroni's post hoc test. *, †, §, and ¶ indicate significantly different from untreated, 5 μg/ml PK, 10 μg/ml PK, and 20 μg/ml PK treated mitochondria, respectively (*p* < 0.05). IMM, inner mitochondrial membrane; Mb, myoglobin; Mic60/Mitofilin, MICOS complex subunit Mic60; OMM, outer mitochondrial membrane; PDH, pyruvate dehydrogenase; PK, proteinase K; and Tom, translocase of outer mitochondrial membrane.

It is necessary to examine the presence of Mb in mitochondria without proteins on the OMM surface in order to clarify if Mb is completely integrated into the mitochondria. PK is a protease that cannot penetrate the intact OMM and IMM under isotonic conditions (van Vlies et al., 2007). It is assumed that the PK treatment can degrade the proteins on the OMM surface without disrupting the isolated mitochondrial structure. Therefore, in this experiment, isolated mitochondria were treated with different PK concentrations to ascertain whether Mb is integrated into the muscle mitochondria. Images of the immunoreactivity for all analyzed proteins are shown in Figure 2a, and the quantification data of the immunoreactivities are shown in Figure 2b–e.

At PK concentrations of 5–10 μg/ml, Mb immunoreactivity showed no significant change compared to untreated mitochondria. At PK concentrations of 20–40 μg/ml, the Mb immunoreactivity was significantly reduced compared to untreated mitochondria (*p* < 0.05) and the immunoreactivity was maintained at more than 68.4% (Figure 2a,b). At a PK concentration of 80 μg/ml, the Mb immunoreactivity was significantly reduced compared to untreated, 5 μg/ml PK and 10 μg/ml PK treated mitochondria (*p* < 0.05), with an immunoreactivity of 59.4% (Figure 2a,b).

Tom20, which has a large cytosolic domain, was used as an OMM protein marker. At a PK concentration of 5 μg/ml, the Tom20 immunoreactivity was significantly reduced compared to untreated mitochondria (*p* < 0.05), and the immunoreactivity was 38.7% (Figure 2a,c). The Tom20 immunoreactivity at a PK concentration of 10 μg/ml was significantly reduced compared to untreated mitochondria and 5 μg/ml PK treated mitochondria, and the immunoreactivity was 27.5% (Figure 2a,c). At PK concentrations of 20–80 μg/ml, the Tom20 immunoreactivity was significantly reduced compared to untreated mitochondria and 5 μg/ml PK and 10 μg/ml PK treated mitochondria (*p* < 0.05), and the immunoreactivity was <8.9% (Figure 2a,c).

Mic60/Mitofilin, which has a large IMS domain, was used as an IMM protein marker. The Mic60/Mitofilin immunoreactivity decreased with PK treatment, although these changes did not reach significance (Figure 2a,d). However, Mic60/Mitofilin immunoreactivities were maintained at more than 51.3% (Figure 2a,d).

PDH was used as a matrix protein marker. The PDH immunoreactivity showed no significant change at PK concentrations of 0–20 μg/ml, whereas the PDH immunoreactivity at PK concentrations of 40–80 μg/ml was significantly reduced compared to 10 μg/ml PK and 20 μg/ml PK treated mitochondria (*p* < 0.05); however, the mean value was higher than 76.5% (Figure 2a,e).

In summary, these data demonstrate that the proteins located on the OMM surface (Tom20) are almost completely digested by PK treatment; however, Mb, IMM protein (Mic60/Mitofilin), and matrix protein (PDH) are resistant to PK treatment. Thus, it is assumed that Mb is truly integrated into the muscle mitochondria.

### 3.2 Mitochondrial protease protection assay

**FIGURE 3:**
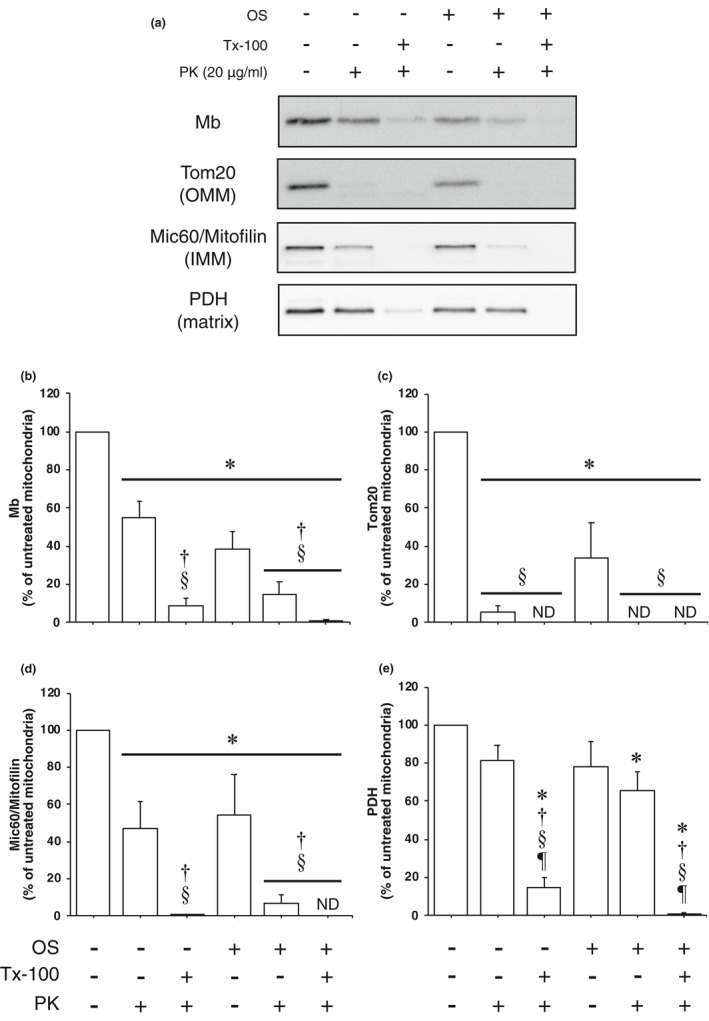
Mitochondrial protease protection assay. (a) Western blotting was performed with antibodies for Mb, Tom20, Mic60/Mitofilin and PDH on isolated mitochondria treated with OS, Tx‐100 and/or PK. (b–e) Quantification of the immunoreactivities of all analyzed proteins in PK treated mitochondria (*n = *3 from separate rats). The immunoreactivities of proteins of untreated mitochondria were set as 100%. The values are means ± SD. Significant differences were assessed using one‐way ANOVA and Bonferroni's post hoc test. *, †, §, and ¶ indicate significantly different from untreated, PK treated, OS treated, and OS + PK treated mitochondria, respectively (*p* < 0.05). IMM, inner mitochondrial membrane; Mb, myoglobin; Mic60/Mitofilin, MICOS complex subunit Mic60; OMM, outer mitochondrial membrane; OS, osmotic shock; PDH, pyruvate dehydrogenase; PK, proteinase K; Tom, translocase of outer mitochondrial membrane; and Tx‐100, Triton X‐100.

Based on the previous results, we performed mitochondrial protease protection assays to determine whether Mb is present in either the IMS side or the matrix side of the IMM. OS treatment is the technique of causing a disruption of the OMM. It is hypothesized that Mb is almost disappeared when the mitoplast is treated with PK (OS + PK treatment) if Mb is localized in the IMS side of the IMM. Images of the immunoreactivity for all analyzed proteins are shown in Figure 3a, and the quantification data of the immunoreactivities are shown in Figure 3b–e.

The Mb immunoreactivity in PK, Tx‐100 + PK, OS, OS + PK, and OS + Tx‐100 + PK treated mitochondria was significantly reduced compared to untreated mitochondria (Figure 3a,b; *p* < 0.05). The Mb immunoreactivity in Tx‐100 + PK, OS + PK, and OS + Tx‐100 + PK treated mitochondria was significantly reduced compared to PK treated mitochondria (Figure 3a,b; *p* < 0.05). The Mb immunoreactivity in Tx‐100 + PK, OS + PK, and OS + Tx‐100 + PK treated mitochondria was significantly reduced compared to OS treated mitochondria (Figure 3a,b; *p* < 0.05). Importantly, The Mb immunoreactivity was almost completely eliminated by Tx‐100+PK, OS+PK, and OS+Tx‐100+PK treatment (< 14.5%) (Figure 3a,b).

The Tom20 immunoreactivity in PK, Tx‐100 + PK, OS, OS + PK, and OS + Tx‐100 + PK treated mitochondria was significantly reduced compared to untreated mitochondria (Figure 3a,c; *p* < 0.05). The Tom20 immunoreactivity in PK, Tx‐100 + PK, OS + PK, and OS + Tx‐100 + PK treated mitochondria was significantly reduced compared to OS treated mitochondria (Figure 3a,c; *p* < 0.05). The Tom20 immunoreactivity was almost completely eliminated by PK, Tx‐100 + PK, OS + PK, and OS + Tx‐100 + PK treatment (<5.7%) (Figure 3a,c).

The Mic60/Mitofilin immunoreactivity in PK, Tx‐100 + PK, OS, OS + PK, and OS + Tx‐100 + PK treated mitochondria was significantly reduced compared to untreated mitochondria (Figure 3a,d; *p* < 0.05). The Mic60/Mitofilin immunoreactivity in Tx‐100 + PK, OS + PK, and OS + Tx‐100 + PK treated mitochondria was significantly reduced compared to PK treated mitochondria (Figure 3a,d; *p* < 0.05). The Mic60/Mitofilin immunoreactivity in Tx‐100 + PK, OS + PK, and OS + Tx‐100 + PK treated mitochondria was significantly reduced compared to OS treated mitochondria (Figure 3a,d; *p* < 0.05). These results are similar to those for Mb, and the Mic60/Mitofilin immunoreactivity was also almost completely eliminated by Tx‐100 + PK, OS + PK, and OS + Tx‐100 + PK treatment (<6.6%) (Figure 3a,d).

The PDH immunoreactivity in OS + PK treated mitochondria was significantly reduced compared to untreated mitochondria (Figure 3a,e; *p* < 0.05). The PDH immunoreactivity in Tx‐100 + PK and OS + Tx‐100 + PK treated mitochondria was significantly reduced compared to untreated, PK, OS, and OS+PK treated mitochondria (Figure 3a,e; *p* < 0.05). The PDH immunoreactivity was almost completely eliminated by Tx‐100 + PK and OS + Tx‐100 + PK treatment (<14.7%) (Figure 3a,e).

In summary, these data demonstrate that Mb, as well as Mic60/Mitofilin, is almost eliminated by OS + PK treatment, suggesting that Mb is localized in the IMS side of the IMM.

### 3.3 Presence of Mb in the supernatant and pellet fractions following OS treatment

**FIGURE 4:**
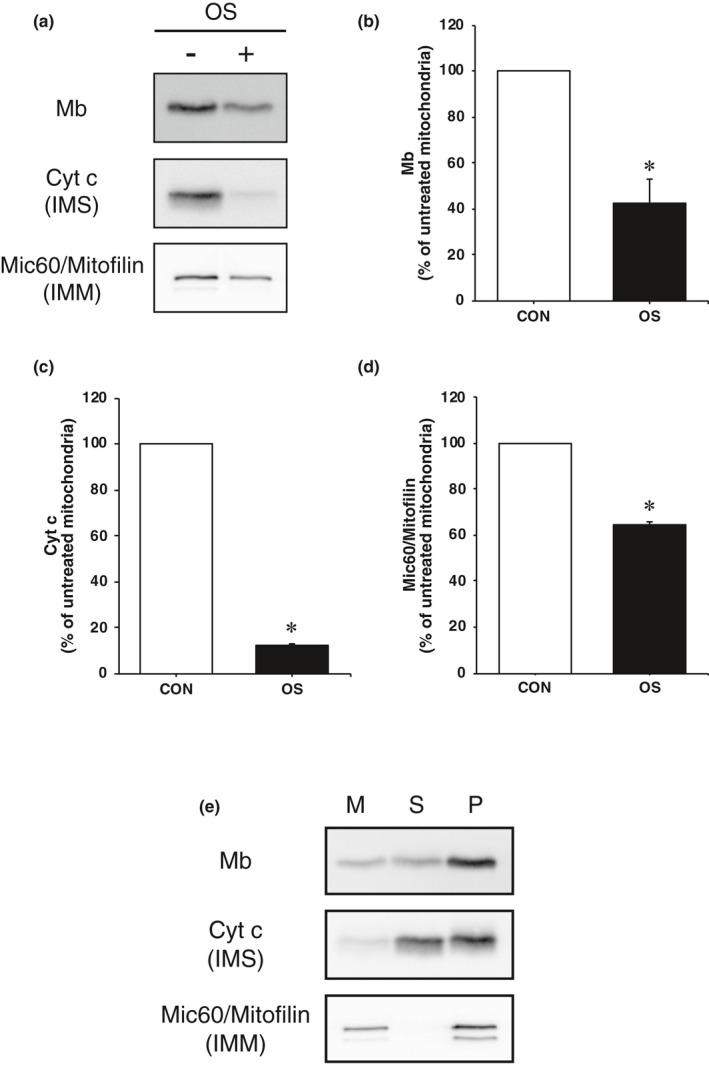
Mb is partially unanchored in the IMS. (a) Western blotting was performed with antibodies for Mb, Cyt c, and Mic60/Mitofilin on untreated and OS treated mitochondria. (b–d) Quantification of the immunoreactivities of all analyzed proteins in PK treated mitochondria (*n* = 3 from separate rats). The immunoreactivities of proteins of untreated mitochondria were set as 100%. The values are means ± SD. Significant differences were assesses using an unpaired *t* test. * indicates significantly different from untreated mitochondria (*p* < 0.05). (e) Presence of Mb in the supernatant and pellet fractions following OS treatment. Cyt c, cytochrome c; IMM, inner mitochondrial membrane; IMS, intermembrane space; M, mitochondria; Mb, myoglobin; Mic60/Mitofilin, MICOS complex subunit Mic60; OS, osmotic shock; P, pellet; and S, supernatant.

We examined whether Mb is partially localized in the IMS using the supernatant and pellet fractions following OS treatment. Mb and Mic60/Mitofilin immunoreactivities in OS treated mitochondria showed significantly reduced values compared to untreated mitochondria (*p* < 0.05), with immunoreactivities of 42.8% and 64.7%, respectively (Figure 4a,b,d). These results are similar to the above results. By contrast, the immunoreactivity of Cyt c in OS treated mitochondria was significantly reduced compared to untreated mitochondria (*p* < 0.05) with an immunoreactivity of 12.1% (Figure 4a,c). Furthermore, after OS treatment, both Mb and Cyt c were detected in both the supernatant and pellet, while Mic60/Mitofilin was detected in the pellet only (Figure 4e). These data suggest that a portion of Mb inside mitochondria is localized in the IMS.

## 4 DISCUSSION

Previous studies suggested that Mb is closely associated with mitochondria (Postnikova et al., 2009; Taylor et al., 2003; Vernier et al., 2007; Yamada et al., 2013). However, whether Mb is integrated into muscle mitochondria remains under debate. Furthermore, the submitochondrial localization of Mb also remains unknown. To the best of our knowledge, this study is the first to report that Mb is truly integrated into the mitochondria of skeletal muscle and that it localizes in the IMM from the IMS side.

### 4.1 Mb is integrated into the mitochondria

Isolated mitochondria were treated with PK to ascertain whether Mb is integrated into the muscle mitochondria. An appropriate concentration of PK cannot penetrate the intact OMM and IMM under isotonic conditions. Therefore, the PK treatment is commonly used as a biochemical approach to ascertain whether a protein localizes inside the mitochondria by digesting proteins on the surface of the OMM (Badugu et al., 2008; Boengler et al., 2009; Lechauve et al., 2012; van Vlies et al., 2007). First, we examined the appropriate concentration of PK to digest proteins on the OMM surface without disrupting the skeletal muscle mitochondrial structure. In this study, we used Tom20 as an OMM marker to confirm whether the PK treatment could digest the proteins located on the OMM surface since Tom20 has a large cytosolic domain. The Tom20 immunoreactivity decreased with increasing PK concentrations, and it was almost completely eliminated at PK concentrations of 20–80 μg/ml, indicating that a PK treatment of 20 μg/ml or more could sufficiently digest proteins located on the OMM surface. The immunoreactivity of Mic60/Mitofilin, which has a large IMS domain, decreased with PK treatment. This suggests that part of the OMM was damaged in the process of mitochondrial isolation; thus, it is speculated that PK penetrated the OMM and a portion of the Mic60/Mitofilin present was degraded. However, the Mic60/Mitofilin immunoreactivity exceeded 51.3% under the PK treatment conditions, demonstrating that at least half of the mitochondria were structurally maintained under PK treatment conditions of 80 μg/ml or less. However, PDH immunoreactivity decreased with 40 μg/ml or more PK treatment, although the immunoreactivity was better preserved (> 76.5%) compared to Mic60/Mitofilin immunoreactivity. This suggests that the PK treatment of 40 μg/ml or more may affect the structure of the mitochondrial matrix. From the results obtained in these experiments, it was decided that 20 μg/ml PK was appropriate to digest the proteins on the OMM surface while minimizing disruption of the mitochondrial structure.

The Mb immunoreactivity at a PK concentration of 20 μg/ml was significantly reduced compared to untreated mitochondria. Several previous studies have reported that Mb is localized on the OMM surface (Postnikova et al., 2009; Taylor et al., 2003; Vernier et al., 2007); thus, Mb on the surface of the OMM was likely digested by PK, thereby decreasing its immunoreactivity. Furthermore, we observed that a portion of Mic60/Mitofilin was degraded by 20 μg/ml PK treatment, suggesting that OMM damage occurs in the process of mitochondrial isolation and that PK may affect proteins inside the mitochondria located at the IMS side. Therefore, it is a possible that Mb inside the mitochondria may be partially digested by PK, thereby decreasing its immunoreactivity. However, the immunoreactivity was well preserved (77.7%), indicating that Mb is integrated into the mitochondria of skeletal muscle.

### 4.2 Submitochondrial localization of Mb

We found that Mb is integrated into the mitochondria of skeletal muscle. Next, we performed mitochondrial protease protection assays using PK, based on a previous study, to investigate the submitochondrial localization of Mb (Badugu et al., 2008). In line with the previous results, the immunoreactivities of Mb and Mic60/Mitofilin in PK treated mitochondria were significantly reduced compared to untreated mitochondria; however, the immunoreactivities were relatively well preserved (Mb: 55.3%, Mic60/Mitofilin: 47.2%). Furthermore, although the Tom20 immunoreactivity in PK treated mitochondria was almost completely eliminated, the PDH immunoreactivity was not changed compared to untreated mitochondria. Moreover, when the PK treatment was performed after dissolving the mitochondrial membranes with Tx‐100, the immunoreactivities of all analyzed proteins containing Mb were greatly reduced. These results indicate that Mb is truly integrated into the mitochondria.

Mitoplasts were obtained by OS treatment to examine whether Mb is localized in the external side or the internal side of the IMM. Tom20 and Mic60/Mitofilin immunoreactivities decreased following OS treatment since not only proteins of the IMS but fragments of the OMM and IMM leak into the supernatant after the first centrifugation (Badugu et al., 2008). In contrast, the PDH immunoreactivity was not significantly changed following OS treatment. Thus, it is assumed that the structure of mitoplasts was not significantly disrupted by OS treatment. Although the Mb immunoreactivity in OS treated mitochondria decreased by approximately 60.0% compared with untreated mitochondria, the immunoreactivity was preserved to some extent (38.7%). However, when mitoplasts were subjected to PK treatment, the immunoreactivities of Mb, Tom20, and Mic60/Mitofilin were greatly reduced as with OS + Tx‐100 + PK treated mitochondria, while the PDH immunoreactivity was similar to that in the OS treated mitochondria. Taken together, these data suggest three possibilities: Mb inside mitochondria localizes in either or both the IMS side of the IMM and OMM. Our previous study has suggested that Mb binds complex IV at the IMM (Yamada et al., 2013); thus, it is plausible that, at a minimum, Mb is localized in the IMM from the IMS side. Since Mb can interact with phospholipids by electrostatic interaction (Basova et al., 2004; Postnikova et al., 2009), the protein may bind complex IV by its association with cardiolipin, a phospholipid contained in the complex by electrostatic interaction (Robinson, 1993).

### 4.3 Mb is partially unanchored in the intermembrane space

Since the Mb immunoreactivity was reduced to some extent by OS treatment, the possibility that a portion of the Mb in the IMS is not anchored and is released into the supernatant by OS treatment has emerged. First, we confirmed that the immunoreactivities of Mb and Mic60/Mitofilin decreased following OS treatment; however, these immunoreactivities were relatively well preserved (Mb: 42.8%, Mic60/Mitofilin: 64.7%). The immunoreactivity of Cyt c was almost completely eliminated by OS treatment. It has been reported that Cyt c, which is an IMS protein, is lost from mitoplasts obtained by OS treatment (Streichman & Avi‐Dor, 1967), implying that in this experiment unanchored Cyt c in the IMS is mostly released into the supernatant. Next, the supernatant collected by centrifugation at 3,000 *g* following OS treatment was subjected to ultra‐centrifugation at 220,000 *g* to confirm the presence of Mb in the supernatant and pellet fractions. The supernatant contains unanchored proteins in the IMS, while the pellet contains fragments of the OMM and IMM. As a result, we found that Mb and Cyt c were detected in both the supernatant and pellet, while Mic60/Mitofilin was only detected in the pellet. Since Cyt c and Mb interact with the IMM, at least under physiological conditions according to previous studies (Cortese et al., 1998; Gorbenko, 1999; Nichols, 1974) and the finding in the present study, these results suggest that a portion of Mb is not anchored in the IMS and bound to the IMM. However, we cannot rule out the possibility that these proteins were aggregated during the ultra‐centrifugation and contaminated the pellet. Further studies are needed to clarify the mechanism for Mb binding to the IMM.

### 4.4 Unresolved questions: The mechanism of Mb import into mitochondria

The mechanism by which Mb is imported into the mitochondria remains unknown, although the present study positively demonstrated that Mb is integrated into mitochondria. Most mitochondrial proteins are produced as precursors on cytosolic ribosomes and transported into mitochondria (Becker et al., 2019; Wiedemann & Pfanner, 2017). The translocase of the outer membrane (TOM) complex is responsible for the initial translocation of approximately 90% of mitochondrial precursors from the cytosol to the IMS. Indeed, connexin 43, which is mainly localized at the sarcolemma, is translocated to the IMS side of the IMM via the TOM complex (Boengler et al., 2009; Rodriguez‐Sinovas et al., 2006). Thus, it is possible that Mb is also imported into mitochondria through the TOM complex. The TOM complex mainly consists of the translocation channel Tom40, and the precursor receptors Tom20 and Tom70. Mb could also be imported into mitochondria through Tom40 with the guidance of Tom20 or Tom70. Further studies are needed to clarify the mechanism of Mb import into mitochondria.

## 5 CONCLUSIONS

The present study showed that Mb is integrated into the mitochondria of skeletal muscle. Furthermore, our data also suggest that Mb localizes in the IMS side of the IMM. Therefore, the possibility that Mb inside mitochondria directly regulate respiration through up‐regulation of complex IV is high (Yamada et al., 2016), although the precise mechanism is unknown. Further studies are needed to clarify the function of Mb inside mitochondria.

## 6 AUTHORSHIP CONTRIBUTION

R.K., K.M., T.O., and T.J. contributed to the conception and design of this study. R.K., T.S., and C.P.L. contributed to the data collection and analysis. R.K., T.S., C.P.L., and K.M., contributed to the data interpretation. R.K., T.S., C.P.L., T.O., H.T., and K.M. contributed to the drafting and critical version of the manuscript. All authors have approved the final version of the manuscript and agree to be accountable for all aspects of the study. All person designated as authors qualify for authorship, and all those who qualify for authorship are listed.

## CONFLICT OF INTEREST

No conflicts of interest to be declared.

7

## References

[phy214769-bib-0001] Badugu, R. , Garcia, M. , Bondada, V. , Joshi, A. , & Geddes, J. W. (2008). N terminus of calpain 1 is a mitochondrial targeting sequence. Journal of Biological Chemistry, 283, 3409–3417. 10.1074/jbc.M706851200 18070881

[phy214769-bib-0002] Basova, L. V. , Tiktopulo, E. I. , Klenin, S. I. , & Bychkova, V. E. (2004). Negatively charged phospholipid vesicles affect tertiary structure of holomyoglobin at neutral pH. Biophysical Journal, 86, 617a.14695306

[phy214769-bib-0003] Becker, T. , Song, J. , & Pfanner, N. (2019). Versatility of preprotein transfer from the cytosol to mitochondria. Trends in Cell Biology, 29, 534–548. 31030976 10.1016/j.tcb.2019.03.007

[phy214769-bib-0004] Boengler, K. , Stahlhofen, S. , van de Sand, A. , Gres, P. , Ruiz‐Meana, M. , Garcia‐Dorado, D. , Heusch, G. , & Schulz, R. (2009). Presence of connexin 43 in subsarcolemmal, but not in interfibrillar cardiomyocyte mitochondria. Basic Research in Cardiology, 104, 141–147. 19242638 10.1007/s00395-009-0007-5

[phy214769-bib-0005] Bradford, M. M. (1976). A rapid and sensitive method for the quantitation of microgram quantities of protein utilizing the principle of protein‐dye binding. Analytical Biochemistry, 72, 248–254. 942051 10.1016/0003-2697(76)90527-3

[phy214769-bib-0007] Cortese, J. D. , Voglino, A. L. , & Hackenbrock, C. R. (1998). Multiple conformations of physiological membrane‐bound cytochrome c. Biochemistry, 37, 6402–6409. 9572857 10.1021/bi9730543

[phy214769-bib-0008] Gorbenko, G. P. (1999). Structure of cytochrome c complexes with phospholipids as revealed by resonance energy transfer. Biochimica Et Biophysica Acta, 1420, 1–13. 10446285 10.1016/s0005-2736(99)00082-6

[phy214769-bib-0009] Hashimoto, T. , Hussien, R. , & Brooks, G. A. (2006). Colocalization of MCT1, CD147, and LDH in mitochondrial inner membrane of L6 muscle cells: evidence of a mitochondrial lactate oxidation complex. American Journal of Physiology‐Endocrinology and Metabolism, 290, E1237–1244. 16434551 10.1152/ajpendo.00594.2005

[phy214769-bib-0010] Izumikawa, K. , Nobe, Y. , Yoshikawa, H. , Ishikawa, H. , Miura, Y. , Nakayama, H. , Nonaka, T. , Hasegawa, M. , Egawa, N. , Inoue, H. , Nishikawa, K. , Yamano, K. , Simpson, R. J. , Taoka, M. , Yamauchi, Y. , Isobe, T. , & Takahashi, N. (2017). TDP‐43 stabilises the processing intermediates of mitochondrial transcripts. Scientific Reports, 7, 7709. 28794432 10.1038/s41598-017-06953-y PMC5550480

[phy214769-bib-0011] Kanatous, S. B. , & Mammen, P. P. (2010). Regulation of myoglobin expression. Journal of Experimental Biology, 213, 2741–2747. 10.1242/jeb.041442 PMC2912755 20675543

[phy214769-bib-0012] Kendrew, J. C. , Bodo, G. , Dintzis, H. M. , Parrish, R. G. , Wyckoff, H. , & Phillips, D. C. (1958). A three‐dimensional model of the myoglobin molecule obtained by x‐ray analysis. Nature, 181, 662–666. 13517261 10.1038/181662a0

[phy214769-bib-0013] Kendrew, J. C. , Dickerson, R. E. , Strandberg, B. E. , Hart, R. G. , Davies, D. R. , Phillips, D. C. , & Shore, V. C. (1960). Structure of myoglobin. A three‐dimensional Fourier synthesis at 2 Å resolution. Nature, 185, 422–427. 18990802 10.1038/185422a0

[phy214769-bib-0014] Lechauve, C. , Augustin, S. , Cwerman‐Thibault, H. , Bouaita, A. , Forster, V. , Célier, C. , Rustin, P. , Marden, M. C. , Sahel, J. A. , & Corral‐Debrinski, M. (2012). Neuroglobin involvement in respiratory chain function and retinal ganglion cell integrity. Biochimica Et Biophysica Acta (BBA) – Molecular Cell Research, 1823(12), 2261–2273. 23036890 10.1016/j.bbamcr.2012.09.009

[phy214769-bib-0015] Nichols, P. (1974). Cytochrome c binding to enzymes and membranes. Biochimica Et Biophysica Acta, 346, 261–310. 4374236 10.1016/0304-4173(74)90003-2

[phy214769-bib-0016] Ordway, G. A. , & Garry, D. J. (2004). Myoglobin: an essential hemoprotein in striated muscle. Journal of Experimental Biology, 2004, 3441–3446. 10.1242/jeb.01172 15339940

[phy214769-bib-0017] Postnikova, G. B. , & Shekhovtsova, E. A. (2018). Myoglobin: oxygen depot or oxygen transporter to mitochondria? A novel mechanism of myoglobin deoxygenation in Cells (review). Biochemistry (Moscow), 83, 168–183. 29618303 10.1134/S0006297918020098

[phy214769-bib-0018] Postnikova, G. B. , Tselikova, S. V. , & Shekhovtsova, E. A. (2009). Myoglobin and mitochondria: oxymyoglobin interacts with mitochondrial membrane during deoxygenation. Biochemistry (Moscow), 74, 1211–1218. 19916935 10.1134/s0006297909110054

[phy214769-bib-0019] Robinson, N. C. (1993). Functional binding of cardiolipin to cytochrome c oxidase. Journal of Bioenergetics and Biomembranes, 25, 153–163. 8389748 10.1007/BF00762857

[phy214769-bib-0020] Rodriguez‐Sinovas, A. , Boengler, K. , Cabestrero, A. , Gres, P. , Morente, M. , Ruiz‐Meana, M. , Konietzka, I. , Miró, E. , Totzeck, A. , Heusch, G. , Schulz, R. , & Garcia‐Dorado, D. (2006). Translocation of connexin 43 to the inner mitochondrial membrane of cardiomyocytes through the heat shock protein 90‐dependent TOM pathway and its importance for cardioprotection. Circulation Research, 99, 93–101. 16741159 10.1161/01.RES.0000230315.56904.de

[phy214769-bib-0021] Streichman, S. , & Avi‐Dor, Y. (1967). The effect of osmotic ‘shock’ on the swelling pattern and respiratory control of rat‐liver mitochondria. The Biochemical Journal, 104, 71–77. 6035525 10.1042/bj1040071 PMC1270546

[phy214769-bib-0022] Tammineni, P. , Anugula, C. , Mohammed, F. , Anjaneyulu, M. , Larner, A. C. , & Sepuri, N. B. (2013). The import of the transcription factor STAT3 into mitochondria depends on GRIM‐19, a component of the electron transport chain. Journal of Biological Chemistry, 288, 4723–4732. 10.1074/jbc.M112.378984 PMC3576077 23271731

[phy214769-bib-0023] Taylor, S. W. , Fahy, E. , Zhang, B. , Glenn, G. M. , Warnock, D. E. , Wiley, S. , Murphy, A. N. , Gaucher, S. P. , Capaldi, R. A. , Gibson, B. W. , & Ghosh, S. S. (2003). Characterization of the human heart mitochondrial proteome. Nature Biotechnology, 21, 281–286. 10.1038/nbt793 12592411

[phy214769-bib-0024] van Vlies, N. , Ofman, R. , Wanders, R. J. , & Vaz, F. M. (2007). Submitochondrial localization of 6‐N‐trimethyllysine dioxygenase ‐ implications for carnitine biosynthesis. FEBS Journal, 274, 5845–5851. 10.1111/j.1742-4658.2007.06108.x 17944936

[phy214769-bib-0025] Vernier, G. , Chenal, A. , Vitrac, H. , Barumandzadhe, R. , Montagner, C. , & Forge, V. (2007). Interactions of apomyoglobin with membranes: mechanisms and effects on heme uptake. Protein Science, 16, 391–400. 17242377 10.1110/ps.062531207 PMC2203327

[phy214769-bib-0026] Wiedemann, N. , & Pfanner, N. (2017). Mitochondrial machineries for protein import and assembly. Annual Review of Biochemistry, 86, 685–714. 10.1146/annurev-biochem-060815-014352 28301740

[phy214769-bib-0027] Yamada, T. , Furuichi, Y. , Takakura, H. , Hashimoto, T. , Hanai, Y. , Jue, T. , & Masuda, K. (2013). Interaction between myoglobin and mitochondria in rat skeletal muscle. Journal of Applied Physiology, 114, 490–497. 23195625 10.1152/japplphysiol.00789.2012

[phy214769-bib-0028] Yamada, T. , Takakura, H. , Jue, T. , Hashimoto, T. , Ishizawa, R. , Furuichi, Y. , Kato, Y. , Iwanaka, N. , & Masuda, K. (2016). Myoglobin and the regulation of mitochondrial respiratory chain complex IV. Journal of Physiology, 594, 483–495. 10.1113/JP270824 PMC4713734 26584944

[phy214769-bib-0029] Yu, Z. , Poppe, J. L. , & Wang, X. (2013). Mitochondrial mechanisms of neuroglobin's neuroprotection. Oxidative Medicine and Cellular Longevity, 2013, 756989. 23634236 10.1155/2013/756989 PMC3619637

